# New York Pathological Society

**Published:** 1859-10

**Authors:** E. Lee Jones

**Affiliations:** Secretary


### ART. III.—*New York Pathological Society.*

REGULAR MEETING, March 27, 1859. JOHN C. DALTON, JR., President,

(Reported for the Journal, by E. LEE JONES, M. D., Secretary.)

Continued from page 231.

### TUMOR OF BREAST.

Dr. A. CLARK presented a specimen of *tumor* of the *breast* removed by Dr. Hyslop. It was sent to him for examination upon the suspicion that it was ordinary scirrhus. There seemed to be no doubt in regard to its character; but, when it came to be examined, it was found to be different from the ordinary cancerous growths, in certain important respects. As, for example,—on cutting into it from the posterior surface about two ounces of gruel-like fluid escaped. A portion of this was put under the microscope, and was found to derive its slight opacity from a world of epithelial cells that were afloat in it. These cells were small, not uniform, and a good deal below the ordinary size of cancer-cells. It struck me, said he, the moment I saw them, that they were a new epithelial structure; and upon further examination I came to the conclusion that it was a cystic growth, and that the greater portion of the cyst was occupied by a firm, fibrous matter, and the portion not so occupied was filled with the fluid referred to. When the solid material was examined, it was found that it was purely fibrous, standing out in papillary eminences. Each one of these prominences, when examined, is found to be covered by the same cells that were found in the gruel-like fluids. As far as he has yet examined the specimen, there seems to be no disturbance in the arrangement of the fibres. He said, the specimen belonged to the class of proliferous cysts, and that it was non-malignant in its character.

Dr. Clark next presented a specimen of *tumor* of the *cerebellum.* The case, said he, from which this specimen was taken, has lately interested us very much at Bellevue. It is that of a woman who died while ether was being administered; and the interesting question came up for solution to-day: whether the ether was the immediate or only cause of death; or whether the diseased mass about to be exhibited was the true cause of death, the administration of ether at this time being no more than a coincidence; and, finally, whether the tumor rendered the patient incapable of bearing the influence of ether, so that the two concurred in the result. In connection with the specimen, Dr. Clark asked the Secretary to read the two following cases of cysts in the cerebellum, from the Liverpool Medico-Chirug. Review, reported by Dr. James Turnbull.

*Case* 1. Samuel Carpenter, a sailor, æt 32, was admitted to the Royal Infirmary on the 12th April, 1857, having been ill with pain in the head and neck, of most intense character, for six weeks. He had the greatest difficulty in turning or moving the head, and the pain was chiefly on the right side. The sight of the right eye was somewhat impaired; but there was no difference in the size of the pupils.

He had weakness, but no loss of power or feeling, in the limbs. The tongue was very much furred when he came in, but it became nearly clean at one time; being variable in its condition. He had occasional vomiting, and the bowels were obstinately confined. Various remedies were tried,—aperients, quinine, and opuim,—without any benefit. It was thought that there might be some deep-seated disease of the vertebræ, or bones at the base of the skull; and issues were made with potassa fusa, and iod. pot. given. These means also failed in affording any relief; and he was then brought gently under the influence of small doses of blue-pill. When his mouth became affected, he appeared to experience great relief; but in a day or two after, he died suddenly whilst eating his dinner. He was admitted on the 12th of April, and died on the 14th of May; having been ill, therefore, about ten weeks.

*Post-mortem.* There was no disease found in the vertebræ of the neck, or in the bones at the base of the skull; but a cyst, the size of a pigeon's egg, was discovered in the right hemisphere of the cerebellum, imbedded in it, and almost entirely covered by the cerebral substance. The walls were very thick, and rather vascular; and it contained a thick, yellowish, clear fluid, with also a clot of blood which occupied about a third of the cyst.

*Case* 2. Mrs. M., a lady about thirty-four years of age, sent for me on the 8th of May to see her, in consultation with Mr. Kay.

The previous summer she had suffered from a severe and acute affection of the brain, for which she had been bled in the arm. She was pregnant at the time, but soon recovered from the attack. Five months previous to my seeing her, she began to suffer severe pain in the head, for which almost every treatment was tried, counter-irritation and depletion, as well as tonics, but all without the slightest permanent benefit. I found that she had very severe pain in the right side of the head behind the ear.

Occasionally, and especially when she moved her head, most acute paroxysms came on, causing her to scream out. At other times she was almost free from pain, but was always rather unwilling to move her head lest it should bring it on. There was great irritability of the stomach, and she frequently vomited dark bilious matter. The bowels were rather costive. The tongue was a good deal furred, especially at the back. There was no heat of skin or febrile disturbance; and the pulse was generally ninety or under. She had deafness of the right ear; but the sight was unimpaired, and there was no loss of sensation or muscular power.

She was brought gently under the influence of small doses of blue-pill; and for a week after, she was comparatively free from pain, so that hopes were entertained of her recovery.

The pain and vomiting then returned as badly as before; and belladonna and Indian hemp were tried, with some advantage.

Morphia, however, was found to afford the most relief.

There was no particular change till the 20th of July; when she had a very severe paroxysm of pain, and died rather suddenly.

*Post-mortem.* On examination of the brain, a cyst was found in the right hemisphere of the cerebellum, about the size of a small hen's egg, and contained a clear, yellowish, serous fluid. The cyst was lined by a very fine membrane, like serous membrane.

The brain was otherwise healthy, and there was no disease of bone.

These cases show us some very important practical facts; and I have not met with any recorded case exactly like them.

They prove that cysts in the cerebellum are at least by no means innocuous, but that they may produce the most intense description of paroxysmal pain; and that *they are probably a more common cause of sudden death than is generally known.*

Dr. E. B. Dalton at Dr. Clark's request, read the history of the case, as follows.

Margaret R——, native of Ireland, single, twenty-one years of age, was admitted into Bellevue Hospital 27th January, 1859. At that time she complained of frequently recurring attacks of intense headache, accompanied with excessive nausea, vomiting, and vertigo. These attacks came on at intervals of four or five days, and lasted some twenty-four or thirty-six hours, and then passed off, leaving the patient comparatively comfortable, though sometimes, still, dull pain in the head and dimness of vision remained. On the closest examination of her previous history and present condition, no clue to the cause of these symptoms could be detected. The bowels were slightly constipated, the urine normal. A physical examination of both chest and abdomen revealed nothing pathological; and, according to the patient's statement, the catamenia had always been normal and she never had sexual intercourse; although the appearance of her breasts, and certain lines upon her hips, gave rise to slight suspicion to former pregnancy. When first admitted, she had been troubled with the symptoms detailed above for some two or three months.

She remained in the hospital until the 4th of the present month (April, 1859) without material benefit. No diagnosis was made. She again entered the hospital on the 7th of April, suffering with the same difficulties unabated. During the attack she apparently suffered the intensest agony, throwing herself from side to side, and at times holding her hands tightly to her head and screaming with pain. Pulse rapid, but not unnatural in force; countenance sometimes pale and sometimes flushed; and the surface generally hot and cold by intervals. The general treatment was alterative in character. During the attacks a great many different things were tried to alleviate the suffering; but the only one known to be successful in the case was the inhalation of sulph. ether, which was entirely so in each instance. The patient would be kept under its influence for several hours. The drug had been administered three times. I gave it myself first, some seven or eight weeks ago, and again two weeks subsequent; the third time, it was given by my assistant. Some two or three ounces were made use of each time; no unpleasant effects had ever been noticed. On Sunday afternoon (April 24th), patient expressed herself as feeling unusually well. On the morning of the 25th, she was found suffering intensely with an attack similar to her previous ones. Ether, at that time, was administered by my assistant. A few minutes afterwards, I was summoned to the ward, where I found the doctor producing artificial respiration. At that time the countenance of the patient was slightly livid, pulse rapid, but of tolerable strength. All voluntary effort at respiration had ceased. She was immediately carried to the window, laid upon the floor, and artificial respiration continued for seven hours. Other means were also used, such as dashing hot and cold water on the body, electricity, ammonia, and injections of hot brandy and water. The pulse remained fairly perceptible for twenty minutes; the hue of the countenance improved at first, then became more livid, and alternated slightly in this way for several hours. At the end of six hours, dark blood began to trickle from the mouth and nose; and at the end of the time above specified, efforts at resuscitation were given up as useless.

*Post-mortem.* Fifty-one hours after death. Body well nourished, upper portions pale, dependent portions somewhat livid; rigor mortis obvious, not extreme; skin in iliac fossæ very blue; nails, and fingers as far as second joint, of same hue; reddish blotches on inner side of lower extremities, and on the interior of right leg.

*Head.* On removing the scalp no unusual appearance is presented, the blood which escaped being of a normal color; skull of average thickness, presenting no peculiarity; surface of brain as seen through dura mater, natural; longitudinal sinus contains a notable quantity of air; surface of brain after removal of dura mater presents nothing abnormal, save the appearance of several bubbles of air in the veins near the longitudinal sinus; surface of brain more dry than usual. On incision of the hemispheres, puncta vasculosa very faintly perceptible; ventricles distended with perfectly transparent serum, an ounce and one-half in quantity. Lining membranes of ventricles perfectly natural in appearance. The remainder of the cerebrum, upon examination appears perfectly natural. *In the cerebellum, nearly in the middle line of the body, is a tumor.* No odor of ether perceptible in the mouth. *Chest.*—About one-eighth inch of subcutaneous fat on the walls of the chest. *Heart.*—Weight, nine ounces; contains perfectly fluid black blood; is well covered with fat, especially the right ventricle, where there appears but little healthy muscular fibre. Left ventricle somewhat contracted, more natural in appearance; slight translucent deposit on the edges of the mitral valve, not sufficient, apparently, to interfere with their function; aortic valves healthy. No disease of valves of right side. Thickness of walls of right ventricle does not anywhere exceed two lines. *Lungs.*—Have traces of old interlobar pleurisy; present nothing abnormal. *Abdomen*, half an inch of fat on walls. *Liver* normal. *Kidneys* both natural, in size and external appearance; on section, intensely congested, especially in the tubular portion. *Stomach* contains a small amount of bloody mucus; mucous membrane of claret color, and covered with small patches of ecchymosis; rugæ very distinct. *Intestines.*—Mucous membrane intensely congested. *Uterus* natural. *Bladder* contains small amount of urine. *Blood* throughout the body fluid and dark-colored.

Dr. Clark remarked, Here is the tumor spoken of. It will be remembered that the surface of the brain was dry; that there was no increase in the thickness of the lining membrane of the ventricle, and the ventricles themselves contained about an ounce and one-half of perfectly transparent fluid; that nothing further was noticed abnormal until the section came down to the cerebellum, when the first slice exposed a portion of this jelly-form material.

This morbid deposit occupied only the left lobe.

The tumor measures three inches in length, two and a half in width, and three quarters of an inch in thickness.

Its character has not yet been ascertained. Some compared it to a large oyster. We have not yet cut into it: it was thought best to exhibit it exactly in this form, and afterwards to make further exploration.

Now then, the question presents itself: Is this really the first case or one of the first cases of mortal issue from etherization; or did the death occur in consequence of the presence of this tumor, which is in the same position and a great deal larger than in the fatal cases reported; or did the existence of this tumor in the position seen, render it more dangerous to administer ether?

Dr. Elliot had charge of the patient during the month of March, and stated that nausea was a very prominent symptom. He referred to that fact, because that symptom was noticed particularly in the cases read by the Secretary.

### Regular Meeting, May 11, 1859.

Dr. Sands, in relation to Dr. Bauer's specimen of cancer of the heart presented the meeting before, stated that he had examined the masses from the endocardium, in connection with Dr. Krakowizer. They presented unmistakable evidences of cancer.

Dr. Bauer makes the following statement in relation to it:—

In order to determine the pathologico-anatomical character of those tumors of the heart, I have subsequently examined them by the microscope, and have found them to consist, but in a small proportion, of encephaloid material, and in the greater, of coagulated fibrin and blood corpuscles. It furthermore seems to me as if the tumors were but loosely connected with the wall of the heart by a sort of adhesive substance. If this be so, then the tumors have been directly formed from the blood by precipitation, and the cancer-cells have been free in the circulation. Unfortunately, the blood has not been examined, and my conjecture remains therefore doubtful. Although, the presence of cancer-cells in the blood is by no means novel, having been observed therein by Virchow, Bennet, Paget, Queckett, Julius Vagel and others: Prof. Bamberger (*Oestreichische Zeitschrift für prakt. Heilk:* 1857, pag. 8) states even that he has observed hepatic encephaloid, which at once would show the modus by which the cancer-cells may enter the circulation.

I have since been informed that Dr. Krakowizer has also examined a specimen of the cardiac tumors, and has substantially attained the same result.

Dr. Finnell presented several specimens of perforation of the ileum of a man who, thirty hours before his death, had been punched in the abdomen and beaten with stones. The peritoneal cavity contained about a quart of serum, and the intestines were adherent throughout the whole of their extent. The rupture is situated midway between the commencement of the small intestine and caput coli. There were no evidences of inflammatory action, around the point of rupture.

Dr. Dalton referred to the case of a boy in the practice of his uncle, who suffered a perforation of the small intestine from the kick of a horse.

Dr. Finnell had presented three such cases.

Dr. Voss referred to a case where rupture of the colon was produced by a blow from the fist.

### DEATH FROM WOUND OF PERICARDIUM AND CORONARY ARTERY.

Dr. Finnell presented, for Dr. Ferguson, a specimen of punctured wound of the heart, with the following history.

William McElroy, aged twelve years and six months, had an altercation with a boy who worked with him at the leather manufactory of Mr. Stevens, in West 27th Street, between 9th and 10th Avenues, during which the boy McElroy was stabbed with a small stitching-awl, over the region of the heart. This occurrence took place on the 17th day of last March, on the fourth story of said building, about two o'clock in the afternoon. A few minutes after being stabbed, the boy reeled over and fainted. He was placed in a recumbent position on a bench. After a lapse of about twenty-five or thirty seconds, he showed symptoms of resuscitation ; and on opening his eyes asked for a drink. For two hours he remained lying down, complaining of great prostration and uneasiness about his heart. A little after four o'clock, feeling relieved, he got up and walked home, which was at the corner of 29th Street and 10th Avenue, on the fourth floor of the building. His mother, seeing him looking very pale and faint, inquired what was the matter. He told her that he was stabbed, and felt very weak and sleepy. He went to bed, but could not lie down, owing to the distress he felt about his heart, with a sense of smothering. He was placed in a rocking-chair, and supported with pillows. Shortly after, he commenced raving and vomiting. He continued so that night, constantly retching and throwing up whatever he drank. His thirst was very great. The next morning he got a quieting powder from a physician, which did not arrest the vomiting, or quiet his mind. In the afternoon of that day he got a few wineglassfuls of an infusion of senna, which he retained, and during that night had a free discharge from the bowels. The next day (Saturday) he was more rational; vomiting ceased, pain about the heart still continuing, with headache; but was enabled to assume the recumbent position occasionally during that day and night. The following day (Sunday) he felt much better; wanted an egg during the morning; sat up during the day; talked rationally; thirst entirely abated, headache gone. Some slight distress still continuing about the heart. He lay down that night without any inconvenience. Monday, much in the same condition. Tuesday he seemed and felt so well that he went to the workshop about nine o'clock, suffering apparently little or no distress from pain or shortness of breath, though he had to descend three steep flights of stairs, and walk from 29th Street and 10th Avenue to 27th Street and 9th Avenue, and ascend three flights of stairs to where he worked. He worked but a short time when he began to cry, and said he felt the pain again. He returned home, and remained in the house that day, not feeling as well as during that morning. The following day (Wednesday) he remained at home, and had a slight inclination to vomit during the day, otherwise feeling well. The next day (Thursday), one week after the accident, he felt quite well; went to work early in the morning, ate well, and went down stairs quite lively; came home to dinner, ate very heartily, coming up stairs quite actively, without any seeming distress or pain; went to work after dinner, and returned to supper. Went out about six o'clock in the evening to walk with a friend; had gone as far as 29th Street and 9th Avenue, when he suddenly complained of weakness and drowsiness, and immediately falling on the sideway, expired. Fourteen hours after death I made a post-mortem examination, and found a small punctured wound on the anterior portion of the chest on the left side, about two inches to the left of the mesial line, between the cartilages of the fifth and sixth ribs. On raising the sternum,

I found that this wound had entered the pericardium, which was filled with fluid and recently coagulated blood, and wounded the left coronary artery about one inch above the apex of the heart, without penetrating its substance. The punctured wound through the skin and soft tissues underneath was nearly closed, and the opening through the pericardium entirely so, and could only be traced by the ecchymosis around where the instrument entered.

Dr. CLARK asked if the opening was closed at the P. M. examination.

Dr. FINNELL found the opening at the P. M. examination, as seen here. The pericardium was distended with blood, partly fluid and partly coagulated.

Dr. CLARK. Was the clot uniform in color?

Dr. FINNELL. It was.

Dr. CLARK remarked that it was difficult to see how the wound should have remained open eight days and death not take place. The distention of the pericardium would limit it to a certain extent, but only enough to allow him to breathe. The pericardium distended with fourteen or eighteen ounces of blood would pretty certainly affect his strength.

He thought the hemorrhage occurred from the pushing out of a plug in the artery by some sudden exertion.

Dr. DALTON thought it probable that the wound might penetrate the wall of the ventricle to such an extent only as to leave a very thin septum, which might give way at the end of a week or more, according to the circumstances of the case.

Dr. FINNELL next presented a specimen of a stomach removed from a patient who was poisoned by cyanide of potassium. The patient was a daguerreian artist, and swallowed a piece of the salt as large as the end of the finger. Immediately he cried for water; but before he could get his mouth to the pipe of the hydrant, he died. Death took place in from three to five minutes after he swallowed the poison.

In answer to a question from Dr. Clark, he stated that the symptoms of poisoning by this salt were very like those from poisoning by prussic acid. The death was very rapid. This was the third case he had met with. This man lived but three minutes; another lived twelve; and a third, he was not certain how long he survived. It was a very short time, however.

In each of the cases the stomach was intensely reddened.

Dr. DALTON thought it was important to know that injection of the stomach took place in so short a time as three minutes, unless most of the change was post mortem.

### FRACTURE AND DEPRESSION OF SKULL.

Dr. Finnell presented a fourth specimen, of a portion of the skull removed from a patient sixty years of age. The man had been stoned by some party, and, as the result, had several scalp wounds. He suffered from more or less concussion, but was soon able to get about. During all this time, however, he complained of a good deal of pain in the head. This state of things continued until the tenth day after the injury, when tetanus showed itself, and he died at the end of the second week.

Post-mortem examination.—One of the scalp wounds corresponded to a point of fracture of the skull with depression; and a portion of stone was found embedded in the skull. The piece of stone was about the size and shape of the semilunar bone. On looking at the internal surface, the depressed bone will be seen with extravasation around it, and a sharp portion projecting down upon the brain.

Dr. Hinton referred in this connection to a patient of the New-York Hospital, who was admitted May, 1856, with a scalp wound caused by a blow from a brick.

The patient did very well until a month after, when unpleasant symptoms began to show themselves, and the parts were thoroughly examined by Dr. Buck. He discovered portions of the brick embedded in the substance of the skull. He removed a button of bone, and there was found a considerable depression of the inner table. The patient died at the end of six or eight weeks after, with purulent absorption.

Dr. Henshell referred to a case of a child who was thrown from a waggon, striking her head against a tree. At the time, nothing was noted but a scalp wound. At the end of about ten days after, a large piece of the bark of the tree was discovered imbedded in the skull. This piece of foreign matter was removed, and the child died the same day.

Dr. Clark, in relation to the case of tumor of cerebellum presented at the last meeting, stated that there were one or two points omitted in the history then read, which he wished to supply by reading the following:

For the last four or five weeks of the patient's life, there was a constant unsteadiness in her gait, and also a marked irregularity in the movements of her hands, when engaged in any occupation. She frequently rolled out of bed; and I find, on strict inquiry, that although she occupied beds in different positions, she invariably rolled out towards her left side. On the morning of her death, she would have once or twice rolled out upon the floor, had she not been prevented by the other patients near her. She was in the habit of walking upon the verandah, and out into the hospital yard, but always with the same staggering, uncertain gait. The general inaccuracy of her movements became so much increased during the last week of her life, that she was unable to sew, according to her previous habits. The nurse reported to me several times that the patient was becoming silly and childish; though, in my own conversations with her, I never noticed anything more than a general sluggishness of speech and manner, which, indeed, had characterized her from the first. The countenance bore a dull, stupid expression. Double vision was a marked symptom, and the right eye was so much impaired in its function as to be nearly useless. The patient was in the habit of covering it when wishing to look at any object.

(Signed) E. B. DALTON, M. D.

These points, said Dr. Clark, are interesting, as bringing the case into the category of those that have been previously recognized. It is true that this was not diagnosticated. At the same time he supposes, that if all these facts had been appreciated as sufficiently before death, as we were induced to appreciate them in the post-mortem, a correct opinion would have been arrived at.

The tumor in the right lobe of the cerebellum was not a cyst. It is uniform in its structure from center to circumference, jellyform in consistence. It would roll about when placed at different inclinations; was semi-transparent, and composed of a hyaline material. In this hyaline material were worlds of little cells, some of which had nuclei of considerable size, others, smaller nuclei. At first view, the cells were so nearly transparent that the mass seemed to be composed of nuclei alone.

The vascularity of the tumor was pretty abundant, though he could discover no one point where a vessel could enter, except it might be at the under surface of the tumor, which was not examined. There may be vascular connection at that point; but that would be the least likely place for it to occur. The vascularity is such as we find in the malignant tumors of the soft variety. In a word, this tumor is of a kind that can get no good name by any fair view that can be taken of it. It as nearly resembles colloid matter as anything we see out of a cyst.

The nerve matter did not enter into its structure at all. The tumor was developed in the white matter of the cerebellum mainly, making a sac of the cortical portion.

In answer to a question from Dr. Markoe, Dr. Clark stated that the tumor was developed in the material of the crus cerebelli, in such a position as to press upon the medulla oblongata, and the inferior point of fifth ventricle, upon the calamus scriptorius.

